# Skin and gut microbiomes of tadpoles vary differently with host and water environment: a short-term experiment using 16S metabarcoding

**DOI:** 10.1038/s41598-023-43340-2

**Published:** 2023-09-28

**Authors:** Bárbara Santos, Filipa M. S. Martins, Joana Sabino-Pinto, Fulvio Licata, Angelica Crottini

**Affiliations:** 1 CIBIO, Centro de Investigação em Biodiversidade e Recursos Genéticos, InBIO Laboratório Associado, Universidade do Porto, Campus de Vairão, 4485-661 Vairão, Portugal; 2 BIOPOLIS Program in Genomics, Biodiversity and Land Planning, CIBIO, Campus de Vairão, 4485-661 Vairão, Portugal; 3 Groningen Institute for Evolutionary Life Sciences, University of Groningen, 9747 AG Groningen, The Netherlands; 4 Departamento de Biologia, Faculdade de Ciências, Universidade do Porto, 4099-002 Porto, Portugal

**Keywords:** Sequencing, PCR-based techniques, Community ecology, Ecological genetics, Freshwater ecology, Microbial ecology

## Abstract

The host-microbiome community is influenced by several host and environmental factors. In order to disentangle the individual effects of host and environment, we performed a laboratory experiment to assess the effects of the exposure to different water sources on the skin and gut microbiome of two amphibian species (*Pelophylax perezi* and *Bufo spinosus*). We observed that the bacterial communities greatly varied with water environment and host identity. Tadpoles of *B. spinosus* collected from a waterbody with poorer bacterial diversity exhibited a more diverse skin and gut microbiome after exposed to a richer water source. Tadpoles of *P. perezi,* originally collected from a richer water environment, exhibited less marked alterations in diversity patterns independently of the water source but showed alterations in gut composition. These results highlight that environment alterations, such as the water source, combined with the host effect, impact the microbiome of amphibian species in different ways; the population history (e.g., previous water environment and habitat) of the host species may also influence future alterations on tadpole microbiome.

## Introduction

Studies on host-microbial associations have shown the important role of microbes in host immunity, physiology, and adaptation, where a poorer or imbalanced microbiome (dysbiosis) is likely to increase host susceptibility to pathogens or disrupt housekeeping physiological functions^1^. These communities have been studied in numerous vertebrate hosts, including amphibians, where particular focus has been given to the skin and gut microbiomes^2^. The intrinsic properties of the amphibian skin (*e.g.*, secretion of peptides and other compounds) influence the community composition^3^, which in turn can influence host’s susceptibility to pathogens^4^. As for the gut, the symbiotic bacteria play a major role in homeostasis, digestive efficiency, and health maintenance^2^. Both skin and gut bacteria communities are affected by biotic and abiotic environmental factors as well as host characteristics^2^. For example, amphibian skin-associated bacteria have been observed to vary according to host identity (*e.g.*, species, ecology, behavior), habitat (*e.g.*, water parameters, soil use, human presence), and climate conditions (*e.g.*, temperature, humidity)^5–7^; gut microbes are often highly associated with dietary preferences, gut mucosal structure, and water source^5,8–10^.

Amphibians typically inhabit freshwater ecosystems, that are among the most threatened yet diverse ecosystems^11^. It is known that alterations in water conditions (*e.g.*, temperature, pH, salinization, pollutants) can alter the environmental bacteria pool, that in turn, can affect amphibian’s microbiome^12^. For example, the microbiome of species that are found in both pristine and anthropized habitats can reflect the different environments^13^.

When a host have contact with a new habitat, it may exhibit a shift in the microbiome composition or function to match the new environment^14^. When contacting with a lower quality habitat, the bacteria pool available to colonize the host often will be reduced, which in turn will lead to a poorer host microbiome when compared with the ones found in more suitable habitats^13,15^. Most studies on the effect of host species or habitat type on microbiome have focus predominantly in the host adult stages^5,15^. However, amphibian early life stages such as tadpoles are highly susceptible to environment alterations, and the study of their microbiome is pivotal to understand the host symbiotic communities at later stages^10^. Moreover, tadpoles are restricted to a single waterbody during the aquatic development phase, and therefore continuously exposed to the same water. The composition of tadpoles’ microbiomes may reveal time-specific dynamics associated with habitat (water) alterations and how the host microbiome responds to external perturbations, turning tadpoles into promising models for monitorization of alteration on waterbodies and its effects of inhabiting populations.

The dynamics of the microbiome community, and the effect of the exposure to new microbial reservoirs may provide new insights on microbe-microbe and host-microbe relationships and on how the host may respond to future environment alterations^12^. Previous studies addressed this habitat effect by translocating individuals to different water bodies in natural environments^14^. In such a complex system it is extremely difficult to exclude the effect of confounding factors. By using a laboratory experiment, we can better control possible confounding factors, and so determine which factor is affecting the bacterial communities. Moreover, it may help to elucidate the timeframe in which community alterations take place, to draw a parallel to observed patterns in nature, and to predict future scenarios^16,17^. Laboratory studies can also provide information on the behavior of symbiotic communities and their effects on the host, that can be useful for other practices such as captive breeding, species conservation, and re-introduction programs^2,18^.

This work explores the simultaneous alterations through time in the bacterial communities of the skin and gut of tadpoles from two amphibian species exposed to the water of their original waterbody (“native”) or to a new water source from a different site (“translocated”) under controlled laboratorial conditions. We aim to analyze how tadpole skin and gut microbiome changes when tadpoles are exposed to different water sources (“native” *vs.* “translocated”) collected from two different waterbodies in laboratory conditions; and if different host species show similar response patterns in their symbiotic communities. Through time, we expected that i) the host identity will greatly influence the symbiotic communities’ composition; ii) the water source will have a significant impact on the tadpoles’ microbiome, with individuals exposed to the new source (“translocated”) experiencing a shift in their microbial community, and that iii) the skin and gut microbiome will show different response patterns.

## Material and methods

### Field sampling

We selected two amphibian species widespread in Portugal, the Iberian green frog *Pelophylax perezi* (López-Seoane 1885) and the European common toad *Bufo spinosus* (Daudin 1803) that can be found in different habitat types, including wetlands, forests, rural and anthropized habitats. Both species can occur in ponds, rivers, and other large waterbodies, with adults of *B. spinosus* usually preferring low-flowing waterbodies (temporary or permanent) and *P. perezi* having a preference for permanent ponds. Tadpoles of both species are generally bottom-dwellers with an omnivorous diet (consuming detritus, algae, plankton, aquatic plants, and arthropods)^19,20^. Tadpoles (at approximately Gosner stage 25;^21^) of both species were collected on day 0 in May 2017. *P. perezi* (n = 66 tadpoles) were collected in Gafanha da Boavista (Aveiro District, Portugal; 40° 36′ 16″ N, 8° 41′ 48″ W), from a stagnant freshwater waterway with an average depth of 60–80 cm, surrounded by agricultural fields. *B. spinosus* (n = 66 tadpoles) were collected in Lousada (Porto District, Portugal; 41° 16′ 23.8″ N 8° 18′ 26.8″ W), from a freshwater lotic stream with an average depth of 30–40 cm, surrounded by vegetation and close-range field pastures. On the same day, 20L of water were collected from each waterbody to fill the aquaria for the experiment. Two swabs from each waterbody were also collected to determine the baseline bacteria community at each water on day 0.

### Experimental design

**Figure 1:**
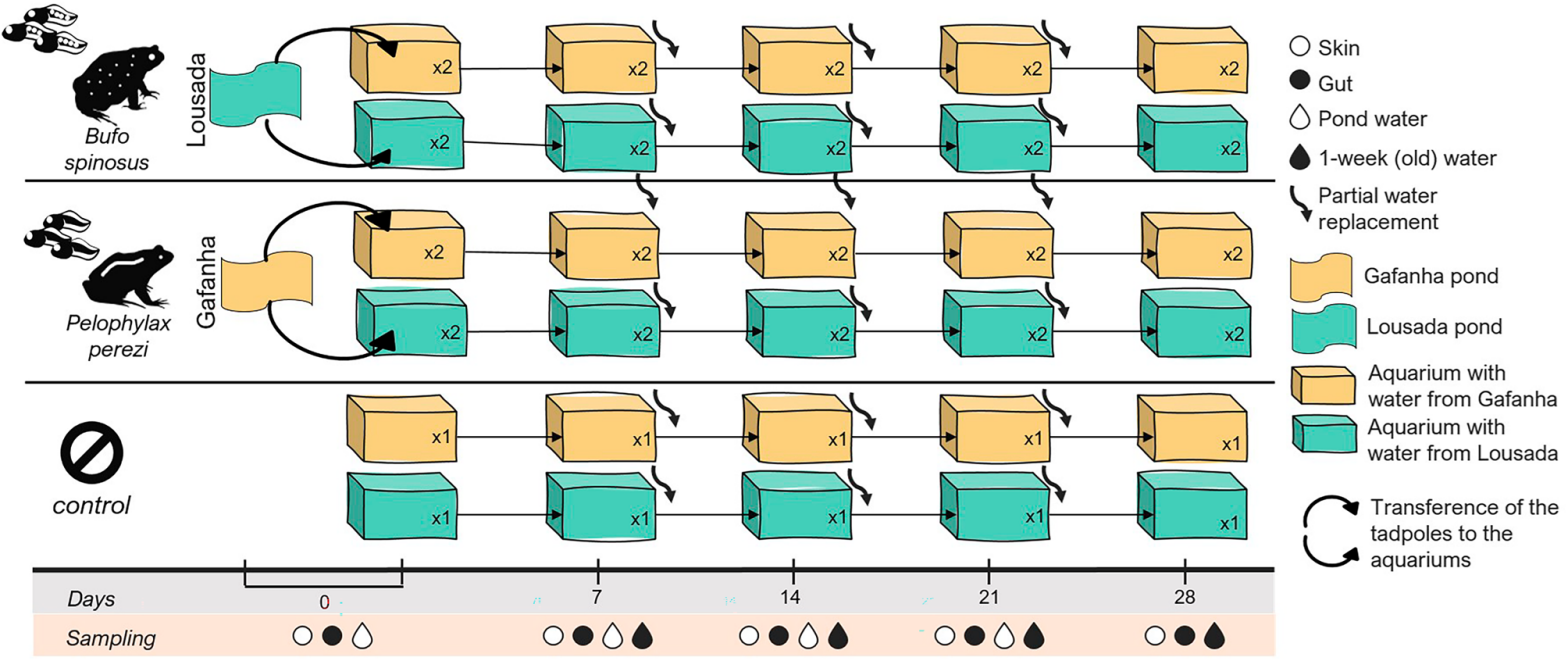
Graphical representation of the 4-week reciprocal translocation experiment carried out in this study using *Pelophylax perezi* and *Bufo spinosus* tadpoles. The experiment design consisted in four experimental groups (2 source waters × 2 host species) and one control group for each source water (aquaria without tadpoles). The figure displays the number of replicas per experimental group (× 1, × 2), the sampling events (Days) and the four types of samples collected at each day – skin (open circle), gut (closed circle), newly collected water from the waterbody (white drop), and water collected from the aquarium (black drop). Different colours represent different water sources: Gafanha (yellow), Lousada (green). Three tadpoles were sampled weekly from each group replicate, where skin swabs and gut were collected. At the sampling days 7, 14 and 21, we partially replaced the water of the aquaria (wave arrow), by mixing freshly-collected water from the waterbody (70%) with the 1-week old water of the respective aquarium (30%), to which tadpoles were exposed in the previous seven days (see Methods for details).

We carried out a 2 × 2 factorial design experiment over four weeks to evaluate the effects of reciprocal translocation on skin and gut bacterial communities of tadpoles of *B. spinosus* and *P. perezi*, exposed to their native or translocated water (Fig. 1). Within each experimental group, two aquaria (replicates) were filled with 4L of water collected from each waterbody, for a total of four aquaria per species (*i.e.*, two aquaria with native water and two with translocated water). On day 0, 15 tadpoles of each species were randomly assigned to one of the four aquaria. All aquaria were maintained under controlled laboratory conditions (12 h light cycle at 24°C) and tadpoles were fed ad libitum with tropical flakes TetraMin® (Tetra, Melle, Germany) every two days. The water of the aquaria was renewed every week (*i.e.*, on days 7, 14, 21) using freshly collected water at both sampling sites. For this renewal, the water in each aquarium was partially replaced by mixing 2,8L of freshly collected water with 1,2L of water where the tadpoles had been in the previous seven days (corresponding to a ratio of 70:30).

### Sampling, amplification and sequencing

On day 0, after collection from the respective waterbodies, six tadpoles from each species were rinsed with sterile water, skin swabbed with a sterile swab, and euthanized in a solution of tricaine methanesulfonate (MS-222, SIGMA) to collect the entire gut. Thereafter, on days 7, 14, 21, 28, three tadpoles per aquaria were collected, skin swabbed, and sacrificed for gut collection. For each experimental group, a total of six tadpoles were collected each week per species per water source (Fig. 1). Samples were stored at  − 20ºC until processing.

DNA was extracted from water, skin swabs and gut using a Qiagen DNeasy Blood and Tissue Kit protocol (Qiagen, Hilden, Germany) with an initial lysozyme incubation step at 37ºC. Bacterial DNA was amplified targeting the V4 region of the 16S rRNA gene with the primer pair 515F (5’-CACGGTCGKCGGCGCCATT-3’) and 806R (5’-GGACTACHVGGGTWTCTAAT-3’). DNA was amplified in duplicate in a volume of 12.5 µl, including 0.2 µl of Phusion Hot Start II DNA Polymerase (Thermo Fisher Scientific, Waltham, Ma, USA), 0.25 µl of each primer (at 10 µM), 0.25 µl of dNTPs, 2.5 µl of buffer, 8.1 µl of H_2_O, and 1 µl of extracted DNA. The amplification protocol consisted of an initial denaturation step at 98ºC for 1 min, 30 cycles of denaturation at 98ºC for 10 s, annealing at 55ºC for 30 s and elongation at 72ºC for 30 s, and a final extension at 72ºC for 5 min. Amplicons of PCR replicates were pooled, visualized on a 1% agarose gel, and purified with QIAQuick Gel Extraction Kit (Qiagen, Hilden, Germany). Samples were sequenced using paired-end 2 × 250 v2 chemistry on an Illumina MiSeq sequencing platform using a dual-index approach^22^ in Plön, Germany.

### Metabarcoding data processing

QIIME2^23^ was used for sequence processing and taxa identification. Due to the typical lower quality of reverse reads^24^, only forward reads were used for the downstream analysis. Sequences were filtered following the criteria: absence of Ns within the sequence, absence of barcode errors, and exclusion of reads containing at least three consecutive low-quality nucleotides. Filtered sequences were clustered into sub-operational taxonomic units (sOTUs, herein called OTUs) following the deblur workflow (https://github.com/biocore/deblur)^25^, trimmed to 150 bp, and excluded OTUs with less than 10 reads. Selected OTUs were taxonomically assigned using Greengenes 13.8 as reference database (May 2013 release; http://greengenes.lbl.gov/) and non-bacterial taxa (*e.g.*, Mitochondria and Chloroplasts) were removed from the dataset. PyNAST^26^ was used to align the OTUs sequences and FastTree^27^ was used to compute a phylogenetic tree. OTUs with less than 0.001% of the total reads from all samples were excluded^28^. A final OTUs table was obtained including all the samples rarefied to 1,700 reads to account for unequal sequencing depth. The microbial dataset was organized into a phyloseq object in R, using reshape and phyloseq packages^29,30^, which included the OTU table, taxonomic annotations of each OTU, metadata (including experimental groups and water source), and a phylogenetic tree.

### Statistical analysis

All statistical analyses and plots were performed in R (Version 3.6.1, R Core Team 2019).

We assessed the variation in skin and gut microbiome diversity and composition of each species at day 0 and exposed to native or translocated water over time. To test our hypothesis, analyses of variance (ANOVA; vegan package^31^) were used to compare the α-diversity (Faith’s phylogenetic diversity) of initial bacteria communities of each host (skin and gut) before the experiment. Generalized Linear Mixed Models (GLMMs; lme4 package^32^) were used to analyze the α-diversity (Faith’s phylogenetic diversity) of bacterial communities (skin and gut) of each species. First, to assess the effect of water source over time, we used a crossed design with “water source” (native / translocated) and “sampling day” (0/7/14/21/28) as fixed effects (interaction only) and the technical replicates (nested within “water source”) as random effects. Second, to evaluate if the overall differences between the two water treatments across time were significant, a post hoc linear hypothesis test (LHT; car package^33^) was performed. Lastly, to evaluate the reliability of the models considering our low sampling size, a power analysis based on Monte Carlo simulations was performed for each GLMM model using the ‘powerSim’ function (‘simr’ R package^34^). The UnWeighted UniFrac distance^35^ was used to build a dissimilarity matrix and permutational Multivariate Analysis of Variance (PERMANOVA; vegan package^31^) were used to analyze β-diversity of bacterial communities using a nested design of three levels–“host species”, “water source” and “sampling day”. To understand the variations in abundance levels, we plotted the 10 most abundant OTUs (at phylum and family levels) in each experiment group across sampling days.

To identify significantly abundant OTUs occurring on skin and gut communities at day 0 and day 28, we performed a Linear Discriminant Analysis Effect Size (LEfSe) analysis with default parameters (LDA score > 2.0, α = 0.05) on the Galaxy web-based interface (http://huttenhower.sph.harvard.edu/galaxy/^36^). We calculated the total unique and shared OTUs for each experimental group within and between species at days 0 and 28. Finally, we calculated the total unique and shared OTUs within each species across the three experimental groups (day0, day28-native, day28-translocated) to understand how many OTUs were constantly associated with each species, meaning that they were not lost after the alteration of the environment and how many OTUs were species-specific. From those, we also identify the OTUs that were consistently associated with all the individuals from each species (core100) in the three experimental groups. All plots were prepared using ggplot2 package^37^.

### Ethical declarations and approval for animal experiments

The sampling was performed with the research permit nº 17105/2017/DRNCN/DGEFF provided by the Institute for Nature Conservation and Forests (ICNF). Only tadpoles were kept in captivity under the protocol approved by the Committee of Animal Experimentation of the University of Porto (Portugal) under the Directive 2010/63/EU of the European Parliament on the 22 of September 2010 and only tadpoles were sacrificed. All methods were performed in accordance with the relevant guidelines and regulations. The experiment was performed complying with the ARRIVE guidelines, such as, providing complete details on guidelines for study design, sample size, experimental procedures, measurements and statistical methods, principle of “3Rs” namely the reduction and reutilization of live animals, among others. The first author has the professional certificate to handle wild animals and perform animal experimentation attributed by national competent authority Direcção-Geral de Alimentação e Veterinária (DGVA).

## Results

On day 0, the skin bacteria communities of *B. spinosus* tadpoles were significantly more diverse than in *P. perezi* (ANOVA, *p* < 0.01), whereas the gut communities were similar between the two species (ANOVA, *p* = 0.942) (Fig. S3).

**Figure 2:**
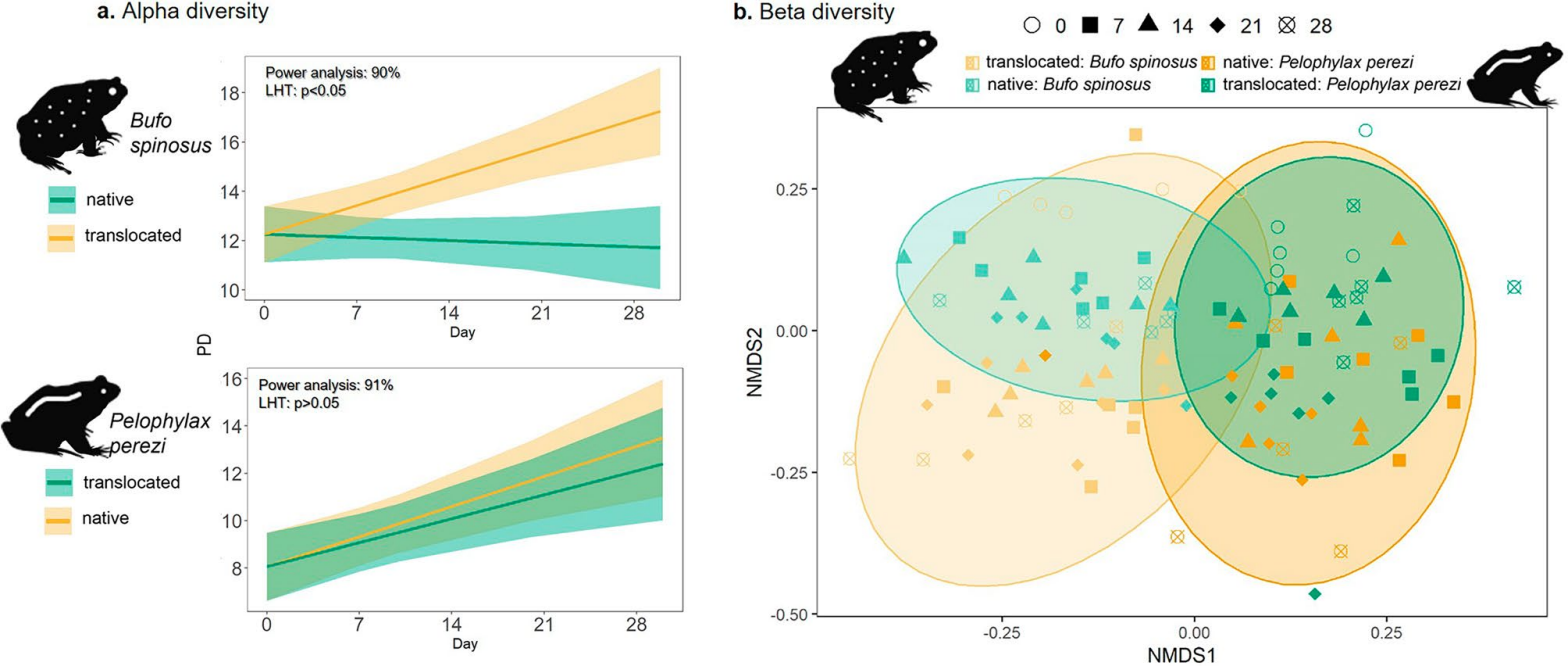
Skin-associated bacterial communities of tadpoles exposed to native and translocated waters sources over time: (**a**) phylogenetic α-diversity detected in each host species, *Bufo spinosus* (top) and *Pelophylax perezi* (bottom); and (**b**) β-diversity (using unweighted Unifrac distances) detected among tadpoles of each species. Yellow and green colour shades represent the water source (Gafanha and Lousada, respectively), ellipses denote the four experimental groups, and symbols indicate sampling events. *P. perezi* tadpoles were collected at Gafanha and *B. spinosus* tadpoles at Lousada, which correspond to their respective native source waters. Values of Linear Hypothesis test (LHT) and Power analysis for each GLMM are indicated within each α-diversity plot.

**Figure 3:**
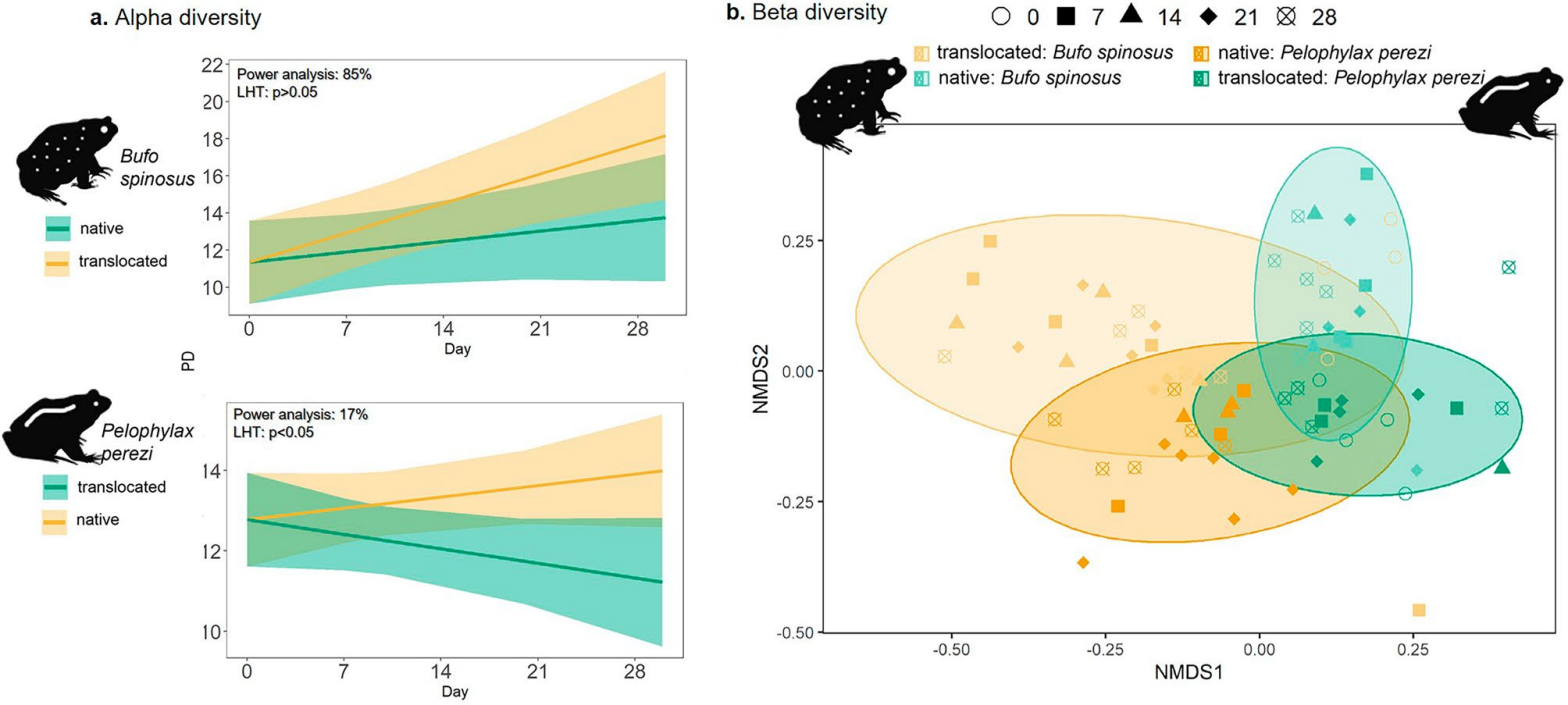
Gut-associated bacterial communities of tadpoles exposed to native and translocated waters sources over time: (**a**) phylogenetic α-diversity detected in each host species, *Bufo spinosus* (top) and *Pelophylax perezi* (bottom); and (**b**) β-diversity (using unweighted Unifrac distances) detected among tadpoles of each species. Yellow and green colour shades represent the water source (Gafanha and Lousada, respectively), ellipses denote the four experimental groups, and symbols indicate sampling events. *P. perezi* tadpoles were collected at Gafanha and *B. spinosus* tadpoles at Lousada, which correspond to their respective native source waters. Values of Linear Hypothesis test (LHT) and Power analysis for each GLMM are indicated within each α-diversity plot.

Over the 28-days experiment, we observed differences between the two host species and tissues (Figs. 2, 3). For the skin communities, *B. spinosus* exhibited, over time, a significant increase of α-diversity when exposed to translocated water (Gafanha) and a stable α-diversity when exposed to native water (Lousada) (Fig. 2a top; Table S1a) with significant differences between the two communities (LHT: χ^2^ = 22.4; *p* = 2.2e−03). For *P. perezi,* there was a significant increase on α-diversity over time in both water sources (Fig. 2a bottom; Table S1b), with no differences between them (LHT: χ^2^ = 0.42; *p* = 0.52). The power analysis supported the reliability of both models (90% and 91% probabilities, Fig. 2a). In gut communities, *B. spinosus* exhibited a significant increase on α-diversity across days when exposed to translocated water (Gafanha), and a stable α-diversity when exposed to native water (Lousada) (Fig. 3a top, Table S1c) but no significant differences were observed between the two water sources (LHT: χ^2^ = 3.46; *p* = 0.06). The power analysis supported the reliability of the model (85% probability). For *P. perezi*, no significant changes were observed in gut α-diversity in neither of the water sources or across time (Fig. 3a bottom; Table S1d), although their α-diversity patterns significantly differed (LHT: χ^2^ = 7.32; *p-value* = 0.006). However, power analysis revealed that this model was inadequate in detecting the tested effect (17% probability).

The variation in β-diversity in skin and gut composition was explained by host identity (8% and 5.9% respectively), water source within host (5% and 10.3%, respectively), and days within water source and host species (8.7% and 10.8%, respectively) (Fig. 2b, 3b; Table S2). The water from Gafanha accounted for higher variation in the composition of skin and gut bacterial communities (inferred by the size of the ellipses) (Fig. 2b, 3b).

**Figure 4:**
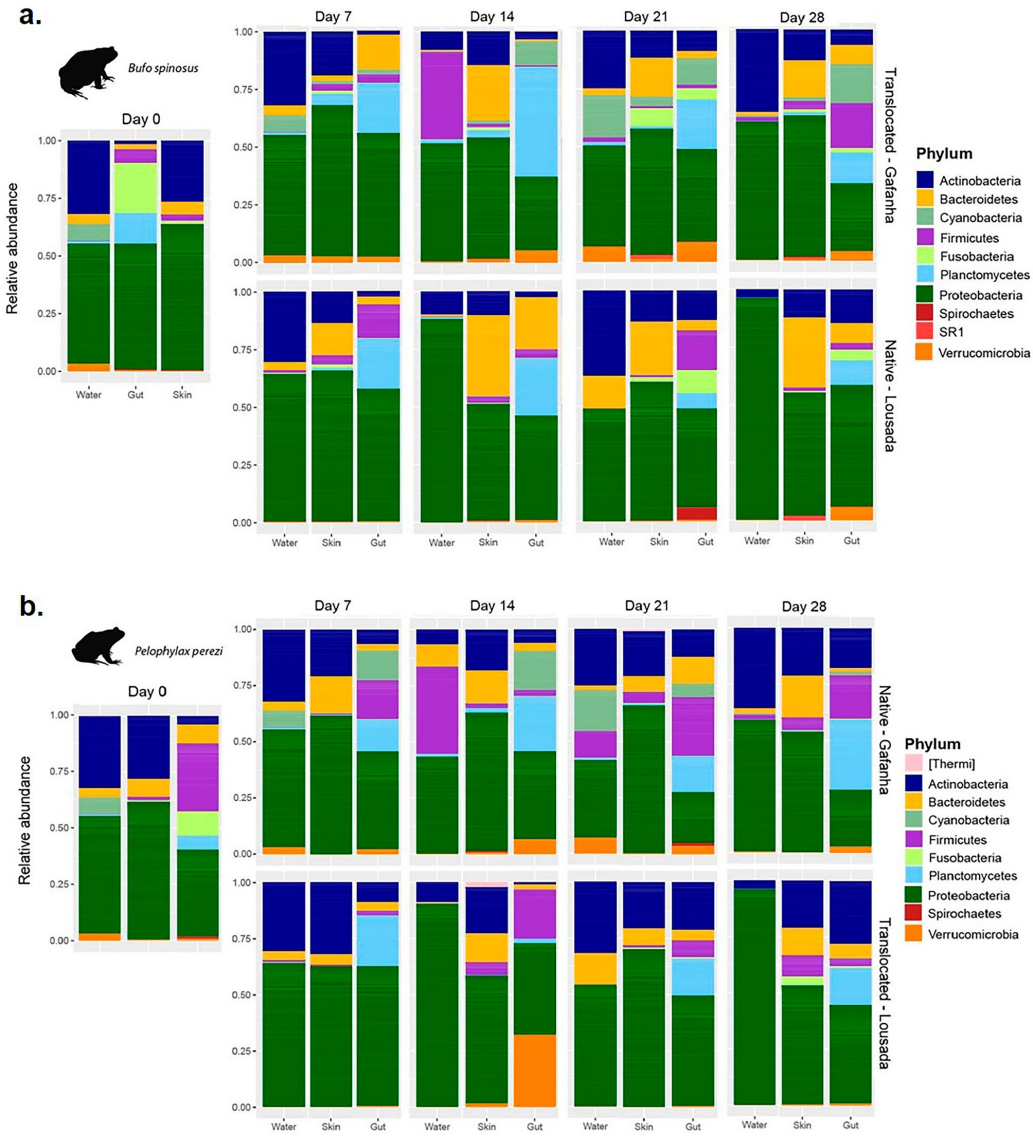
Abundance of the 10 most prevalent bacterial Phyla in the skin and gut communities of (**a**) *Bufo spinosus* and (**b**) *Pelophylax perezi* in comparison with those found in the water where tadpoles were reared at each sampling event (Day).

**Figure 5:**
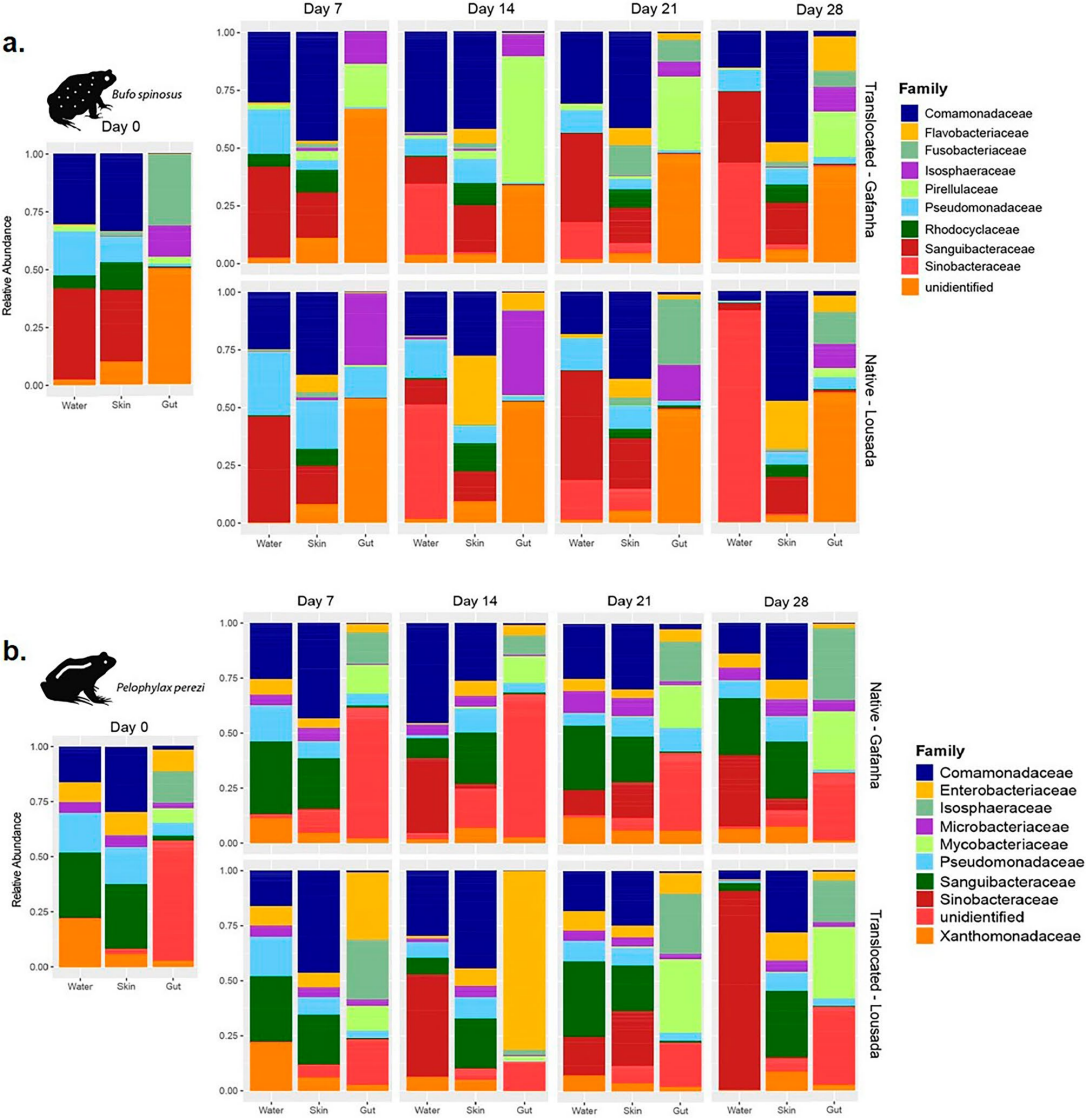
Abundance of the 10 most prevalent bacterial families in the skin and gut communities of (**a**) *Bufo spinosus* and (**b**) *Pelophylax perezi* in comparison with those found in the water where tadpoles were reared at each sampling event (Day).

Gut communities had distinctive abundant bacteria families and higher number of unidentified taxa when compared with skin and water communities (Figs. 4, 5). Actinobacteria and Proteobacteria were the most abundant phylum in the water and skin of both species, while Planctomycetes and Firmicutes were more abundant in the gut (Fig. 4). The two species harbored distinct abundant families on day 0, with *B. spinosus* carrying more Rhodocyclaceae (skin) and Fusobacteraceae and Isosphaeraceae (gut) (Fig. 5a), whereas *P. perezi* had higher abundance of Enterobacteriaceae (skin) and Enterobacteriaceae and Isosphaeraceae (gut) (Fig. 5b). The differences between the two species were consistent across days with Rhodocycladeae being abundant in the skin of *B. spinosus* but not in *P. perezi*.

LEfSe analysis identified 19 differential abundant taxa in the skin and 27 in the gut depending on the species, on the water medium and sampling day (Figs. S4). In the skin, the two species had more differentially abundant taxa at day 0 (Fig. S4) and *P. perezi* had more differentially abundant taxa when exposed to translocated water (Lousada). *P. perezi* tadpoles exposed to native water (Gafanha) exhibited only one significantly abundant taxon, *Nevskia ramose*; whereas *B. spinosus* exhibited one abundant taxon in each water source: *Flavobacterium succinicans* when exposed to native (Lousada) water and *Hydrocarboniphaga effusa* when exposed to translocated (Gafanha) water (Fig. S4).

In the gut, both species showed more than four differential abundant taxa at days 0 and 28 when exposed to water from Lousada, regardless if this was native (in the case of *B. spinosus*) or translocated water (in the case of *P. perezi*) (Fig. S4). At day 0, *B. spinosus* carried high abundance of *Rhodobacter*, which was maintained until day 28 in native water source (Fig. S4).

At the beginning of the experiment (day 0), the hosts shared less than 10% of skin or gut taxa while at the end of the experiment (day 28), each group (host species x water sources) maintained a higher number of unique OTUs (Fig. S5). Overall, hosts exposed to richer water source (Gafanha) harbored more unique skin OTUs and shared more OTUs among them (Fig. S5a,b), pattern also observed for gut communities (Fig. S5c,d). *B. spinosus* carried a higher number (above 10%) of shared OTUs across time (day 0 and 28) on skin and gut communities, while tadpoles of *P. perezi* shared always less than 3% OTUs (Fig. S6).

Tadpoles of *B. spinosus* carry a richer core100 community in the skin (9 OTUs) and gut (6 OTUs), while *P. perezi* show a poorer skin core100 community (2 OTUs) and no gut core100 (Table S3).

## Discussion

Our study explores the effects of the exposure to different water sources on the skin and gut bacteria communities of tadpoles of two amphibian species, *Bufo spinosus* and Pelophylax perezi, collected from different sites and maintained in controlled laboratory conditions over a four-week time period. The two target species did not share the same habitat in the field, so the “water source” factor and the “host identity” factors are intimately linked. Our translocation experiment nevertheless breaks this link on the short term by accompanying the changes across weeks in each species. However, it is reasonable to consider that our “day 0” conditions reflect a kind of optimal equilibrium for the amphibian fitness in their habitat and therefore an expected natural microbiome for each species. Since our experiment was a short-term one, all the following discussion should be seen at the light of this limitation. In particular, our experience does not assume the long-term stability of the new associations, neither the outcome of these new associations on the fitness of their amphibian hosts. Our experiment shows how the current native microbiome (resulted from the combination of host species and habitat at day 0) changes in laboratorial conditions exposed to certain types of water sources in controlled laboratory conditions.

The experiment duration of 28 days was sufficient to unveil significant differences in the symbiotic communities of the two host species which supported our hypotheses showing that: (i) host species has a significant effect in their associated bacteria communities observed across the experiment at laboratorial conditions; (ii) the water source influences the microbiome of both species; and overall, (iii) skin and gut communities respond differently to environmental changes. The way these hypotheses are met is discussed below following the experiment timeline: day 0, exposure period, day 28, to highlight the patterns observed.

The influence of host species on symbiotic communities has been one of the major factors studied^5–7,16,38^. At day 0, *B. spinosus* tadpoles collected from the pond with poorer water bacterial community harbored a more diverse skin bacteria community than *P. perezi* tadpoles collected from a waterbody with richer water bacterial community (Fig. S3A). This highlights the role of the host in filtering bacteria. In previous studies, anurans with more terrestrial behavior, such as toads, have been found to carry richer microbiomes than frogs^38^. This has been associated with terrestrial habits and exposure to pools of bacteria from land and water, but the occurrence of the same pattern in tadpoles indicate a strong effect from other factors, like host genetics, ecology, or skin properties, among other traits^7,39,40^. Ultimately, microbiomes may be transferred across developmental stages and tadpoles with richer microbiomes can develop into adults with richer microbiomes which may aid the host in disease resistance leading to an ecological advantage^41,42^. Contrary to the skin, gut communities of the two species exhibited similar diversity levels but shared few OTUs evidencing again the host effect even if under the same diet. Previous studies have also found significant host-effects on gut bacteria and proposed different gut physiologies and diet as most likely explanations^9,10,14^. The strong host-specificity of microbiomes makes it a challenge to generalize conclusions about the effects of habitat alterations like the one studied here, but on the other hand increase the need for experimentation in a wide range of species. Throughout the experiment, and supporting our first hypothesis, the two species maintained distinct symbiotic communities, even when exposed to the same water source (Figs. 2, 3), a pattern that was in accordance with previous studies on amphibians supporting the host as major factor^5,7^. In the skin, this was especially visible by the high overlap in community composition of intraspecific tadpoles exposed to different waters (Fig. 2), suggesting that each host harbors permanent bacteria or filter different taxa despite of the water source^10,43^. From one side, this can be associated with characteristics of the bacteria (e.g., taxa that are more generalist or tolerant to environmental changes are more likely to be retained within the host tissues)^42^. On the other hand, these symbiotic communities can be filtered by the host due to genetics, ecology/behavior (e.g., aquatic vs terrestrial;^38,40^, physiology, skin structure and mucous can filter different bacterial taxa;^44,45^) among others. Although adults of *B. spinosus* have dry and rugged skin and *P. perezi* have a smooth moisty skin, tadpoles of the two species have similar skin texture and appearance, thus the effect of skin compounds is probably one factor at the larval stage. For example, tadpoles of some bufonids may contain skin noxious compounds to deter predators and can influence microbiome^46^. In the gut, the host effect was also supported throughout the experiment, observed in the community composition exhibiting low overlap between the two species when exposed to the same water source (Fig. S1). At the end of the 28 days, in spite of the two species continuing to exhibit significant distinct communities, they carried less high-abundance OTUs in comparison with day 0. This suggests that there was a restructuration in the bacteria community and that this is probably related with other factors, such as after exposure to different water conditions in the experiment^10,12,14,16^.

Supporting the second hypothesis, the effect of water source was found to influence the bacterial communities in both host species. Previous field studies have also shown that species’ microbiome can vary with surrounding habitat conditions, water bodies with different flow regimes, and other waterbody characteristics (e.g., size, chemical properties, temperature)^47,48^. On day 0, tadpoles of *B. spinosus,* collected from a lotic environment, had a more diverse skin community comparing with *P. perezi*, collected from a stagnant freshwater body. Although here, the waterbody effect cannot be individualized from the host effect, it is highly probable that the freshwater stream from Lousada influences in part the higher microbial diversity of tadpoles in their natural habitat^47^. Throughput the experiment, tadpoles exhibited an increase of skin diversity consistently higher when exposed to the water source from Gafanha site with the richest bacterial pool (Fig. 2). The increase in skin bacteria α-diversity in hosts exposed to a new water source is likely correlated with higher number of colonization events by new bacterial taxa. Tadpoles of *B. spinosus* exhibited a notable increase in skin and gut α-diversity when exposed to richer water source from Gafanha, suggesting that they were able to received more new bacteria than when exposed to the water source from their native habitat^14,48^. The richer community of *B. spinosus* may result also from the combination with retaining taxa from the native environment, but suggests that the microbiome is highly susceptible to new inputs of environmental bacteria which ultimately can be beneficial or damaging depending of the taxa^41,46^. The observed increase in intra-specific tadpoles’ skin microbiome α-diversity when exposed to different water sources was mostly driven by less abundant bacteria. The most common bacteria demonstrated the expectedly strong host effect, although we cannot ascertain whether this is due to host filtering, or the bacteria themselves being generalist^15^. When comparing both species microbiome exposed to the same water source, they exhibited an unexpected strikingly lower gut community overlap (Fig. 3). This lower overlap in the gut microbiome compared to the skin may indicate that the gut microbes are more resilient to environmental changes given the buffered inner-body environment or that the native gut bacteria strongly influence the future colonizers and community reassembly (Fig. S1D)^14,44^. *P. perezi* tadpoles showed smaller variations on skin α-diversity but differences in beta diversity between the two water sources (Fig. 2a,b). This suggests two hypotheses: the first is that tadpoles’ skin were colonized by new bacteria only through replacement of previous taxa, maintaining the total diversity similar when exposed to either water sources; the second is since *P. perezi* tadpoles were collected from a pond within an agricultural area, there is a high probability of carrying a higher functional resident symbiotic community providing resilience and adaptability to cope with alterations in water environments and therefore with limited need or space to recruit more taxa^13,15,45^. When exposed to richer water (Gafanha), individuals from both species shared more skin OTUs among them, than individuals within same species exposed to the poorer water (Lousada). These common OTUs were likely acquired from the richer water environment and may indicate that these taxa have a better capacity of colonization^12,49^. Tadpoles exposed to the richer water source also showed increased α-diversity and, more unique OTUs, but exhibited lower number of highly abundant OTUs, indicating a trade-off between higher diversity and taxa dominance that may be associated with specific community response to the water source. Contrary to that, tadpoles of both species exposed to water with poorer bacteria diversity harbored more highly abundant skin and gut OTUs and fewer unique taxa, suggesting that some OTUs were likely able to increase in abundance to fill the available space in a less diverse skin and gut environment. Finally, at day 28, the taxa abundance was a good indicator of experimental groups (Figs. 4, 5), affirming the water source as a highly significant influence on the symbiotic communities for both species.

Skin and gut microbiomes are the most commonly studied symbiotic communities, yet still rarely evaluated together, and even less so in frameworks comparing species or responses to environment changes^40,50–53^. Supporting our third hypothesis, the skin and gut communities from both species exhibited very different response patterns when exposed to the two water sources. At day 0 and throughout the experiment, the skin community from the two species varied more in terms of α-diversity while gut community exhibited more differences in terms of community composition (Figs. 2, 3), differences associated with the type of community and exposure level. The skin can mediate and filter microbiota (through e.g. thickness, texture, dryness, excretions;^3,4,6^) but is also in direct contact with the surrounding external environment, which means higher rates of contact with new bacteria, and higher potential for fluctuations in colonization rates and diversity levels^17,41^. The gut, on the other side, is an internal environment, that is both more stable and extreme (e.g., in terms of pH), thus creating stronger filters for potential colonizing bacteria from the exterior^13–15,42^.

One possible caveat of this work is that we introduced an artificial diet that can affect both skin and gut microbiotas, often leading to a homogenization effect of the microbiome between host species^54–57^. However, we observe significant differences in both α- and β-diversity between species which suggests that the effect of the diet was lower compared with the two main factors evaluated here: host and water^14,46^.

Another limitation is that laboratory conditions are more stable than the natural environment, which may alter selective pressures on the microbiome, and possibly favor poorer and more homogeneous communities^6,41,58,59^. We attempted to limit this caveat by using a short-term experiment and renewing water every week^60,61^. While diminishing lab-condition effects, this water renewal approach creates the additional factor of the input of new transient bacteria every week^62,63^. However, given the aims of this study were to analyze how different water sources affect amphibian microbiomes, while analyzing host effects, the weekly water renewal was deemed the best option. Moreover, the two species exhibited different α- and β-diversity when exposed to the water sources (same microbial pool) supporting the stronger influence of host identity and water as main factors shaping the skin and gut microbiomes.

## Conclusions

The findings from our study revealed that a short-time experiment is enough to observe both host and water influences on the skin and gut microbiome of tadpoles. At shorter time periods, captivity conditions and diet seem to have a small influence on the bacterial communities of both species. Despite the possible confounding effect of the entanglement of host identity and habitat in the field, the input of water was common to the two species each week (same water source from the same day) and the two species maintained their distinct microbial composition throughout the experiment in both skin and gut communities (especially in terms of beta diversity and unique OTUs) supporting the host identity/origin as a major determinant. The bacterial communities of *Bufo spinosu*s were especially affected by water sources if compared with *Pelophylax perezi*, and were found to greatly vary across time when exposed to a richer water. Future work should compare different symbiotic communities within a host to determine if there are exchanges between specific bacterial taxa (*e.g*., substitution, loss or gain of specific OTUs), assess which are their physiological roles within the host, and test if there is an association with the specific water characteristics. Additionally, studies are needed with more species and water sources to establish response patterns for the skin and gut communities. This should be combined with controlled mesocosms studies addressing more factors combined at a time.

### Supplementary Information

Supplementary Information.

## Data Availability

The dataset of this article is available at the NCBI repository under Bioproject ID PRJNA1014117 through the direct link https://www.ncbi.nlm.nih.gov/bioproject/PRJNA1014117.
