# Supplementary material for: Skin and gut microbiomes of tadpoles vary differently with host and water environment: a short-term experiment using 16S metabarcoding
Source: Sci Rep. 2023 Sep 28;13:16321. doi: 10.1038/s41598-023-43340-2 (PMC10539280; doi:10.1038/s41598-023-43340-2)
Supplement: Supplementary file 1 — Supplementary Information. [file 41598_2023_43340_MOESM1_ESM.docx]

**Supplementary File**

Skin and gut microbiomes of tadpoles vary differently with host and water environment in a short-term experiment using 16S metabarcoding

Bárbara Santos^1,2*^, Filipa M.S. Martins^1,2^, Joana Sabino-Pinto^3^, Fulvio Licata^1,2^, Angelica Crottini^1,2,4^

**Index**

Supplementary Material and Methods ………………………………………………………………… 2

Supplementary Results …………………………………………………………………………………. 3

Supplementary Discussion ……………………………………………………………………………... 3

Supplementary References …………………………………………………………………………….. 4

Supplementary Figures and Tables …………………………………………………………………… 5

**Supplementary Material and Methods**

**Experimental design – controls**

One aquarium filled with water collected from the waterbody from Gafanha and one filled with water collected from the waterbody from Lousada, was maintained throughout the experiment at the same conditions but without tadpoles. These controls were used to compare the bacteria communities in the water sources each week, without the influence of tadpoles, since individuals are also expected to release bacteria into the water environment.

The water bacteria were also characterized in all aquaria including control aquaria (aquaria without tadpoles), aquaria with water collected at Gafanha site and with tadpoles of each species and aquaria with water collected at Lousada site with tadpoles of each species. This sampling was done before changing the water and after mixing with the new water fresh collected at the day from each site. The same sampling of water bacteria was done in each waterbody before transporting to the laboratory to be able to compare after the mixture.

The sampling of the water bacteria included moving a swab for 10 seconds, into the waterbody on days 0, 7, 14 and 21 (Pond water) and from each aquaria (enumerated in the paragraph above) at days 7, 14 and 21.

**Statistical analysis**

The final OTU table included taxa from skin, gut and water samples. For the microbial community of the water samples, some additional steps were performed: i) at day 0, for each waterbody, the read counts obtained from the two swabs were summed and rarified (at 1700) to match the same rarefaction in other samples (skin and gut); ii) for the exposure period, the bacteria community of the “water environment” in each aquarium of each week was obtained considering the mixture ratio 70:30 in the following way: we used a randomized subsampling of 30% of the reads observed in the swab water samples collected at each aquarium (to which tadpoles have been exposed during the previous 7 days) and summed this to a randomized subsampling of 70% of the reads obtained from the newly collected water at the respective waterbody, previously rarified to 1700 reads. From the final dataset, we calculated the phylogenetic diversity of all samples (using *picante* package [Kembel *et. al*. 2010]).

Before testing our hypotheses, we investigated whether there were differences in the bacteria communities between the two water sources from each waterbody (Gafanha and Lousada) at the beginning of the experiment (day 0). Additionally, we characterized the communities of the water in each aquarium (experimental groups) over time in relation to the source (water collected at the ponds) to confirm that the water in the aquaria was similar and representative of the water collected on the same days in the water bodies and that both water treatments (Gafanha and Lousada) had significantly different bacterial communities guaranteeing that tadpoles would be exposed to different bacteria pools. To do this, we explored the variation in bacteria alpha (α) and beta (β) diversities using phylogenetic diversity and Unweighted unifrac distances, respectively, at the water swabs from each waterbody (Gafanha and Lousada) and in the aquaria water mixtures used in the experiment (Fig. 1; Fig. S1). Due to low sampling size of water samples, direct comparisons were performed for α-diversity using a line plot, while non-metric multidimensional scaling was used to plot the β-diversity (Fig. S1). The relative abundance of the ten most abundant taxa (at phylum and family levels) in the water samples were visualized using a barplot, and displayed by water source and sampling event (day) (Fig. S2).

**Supplementary Results**

Analysis of water microbiome in water sources and water from aquaria without tadpoles (control) and with tadpoles

Differences in bacteria phylogenetic diversity and composition were observed between the water sources (Fig. S1), with water collected from Gafanha showing higher α-diversity across all days. Some variability in α-diversity was observed within each collected water throughout time, but the pattern was maintained in the water collected from the experimental groups (e.g., Control, *Bufo spinosus*, *Pelophylax perezi,* Fig. S1a-c). A few exceptions were: Gafanha water collected from the aquaria hosting *B. spinosus* tadpoles at days 7 and 14 and the Lousada water of the aquaria hosting *P. perezi* tadpoles at day 14 (Fig. S1b-c); in these situations, the α-diversity from the experimental groups was higher than the one from the source water. In terms of β-diversity, water samples were consistently grouped by source water and sampling day (Fig. S1d). In terms of composition, the relative abundance of the most prevalent bacteria differed between water sources: both carried high abundance of phyla Proteobacteria and Actinobacteria across the sampling days, but the water collected at Gafanha exhibited high abundance of Cyanobacteria, Firmicutes and Verrucomicrobia, while the water from Lousada exhibited higher abundance of Bacteroidetes; at the family level, water from Gafanha exhibited higher variation in abundance levels over time (Fig. S2a-b).

**Supplementary Discussion**

In order to analyze the water microbiome alterations, the aquaria without tadpoles were used as controls to understand the alterations in bacteria from each water environment without the influence of the tadpoles since hosts may release bacteria into the water. In this way it was possible to understand if the water environment from each experimental group (and after the mixture 70%-30%) was representative of the water in the natural environment on the same day. We confirmed that the aquaria water was a good representation of the freshly collected water at each site (Fig. S1) namely, the water microbiome from aquarium was similar to the water microbiome collected at the waterbody on the same day. We also can be sure that the two species were exposed to similar water bacteria pool when exposed to the same water source over time in the laboratory. Moreover, the water collected at the two waterbodies (Gafanha and Lousada) presented distinct bacteria communities with water from Gafanha being more diverse (consistently throughout the experiment), therefore indicating that tadpoles exposed to this water entered in contact with a more diverse and richer environmental pool of bacteria during the experiment (Fig. S1). The higher bacteria α-diversity observed in Gafanha water could result from the presence of agricultural fields near the sampling site and likely run-offs products from the soils leading to more frequent alterations in water composition. Soils treated with fertilizers or pesticides can exhibit an increase of dissolved organic matter and consequently an increase in the microbial diversity which in turn could affect water environment and amphibian’ microbiome [Chang *et al*., 2016].

Kembel, S. W. *et al*. Picante: R tools for integrating phylogenies and ecology. *Bioinformatics* **26**, 1463–1464. <https://doi.org/10.1093/bioinformatics/btq166> (2010).

Chang, C-W., Huang, B-H., Lin, S-M., Huang, C-L., Liao, P-C. Changes of diet and dominant intestinal microbes in farmland frogs. *BMC Microbiol*. **16,** 1–13. <https://doi.org/10.1186/s12866-016-0660-4> (2016).

**Supplementary Figures and Tables**

**
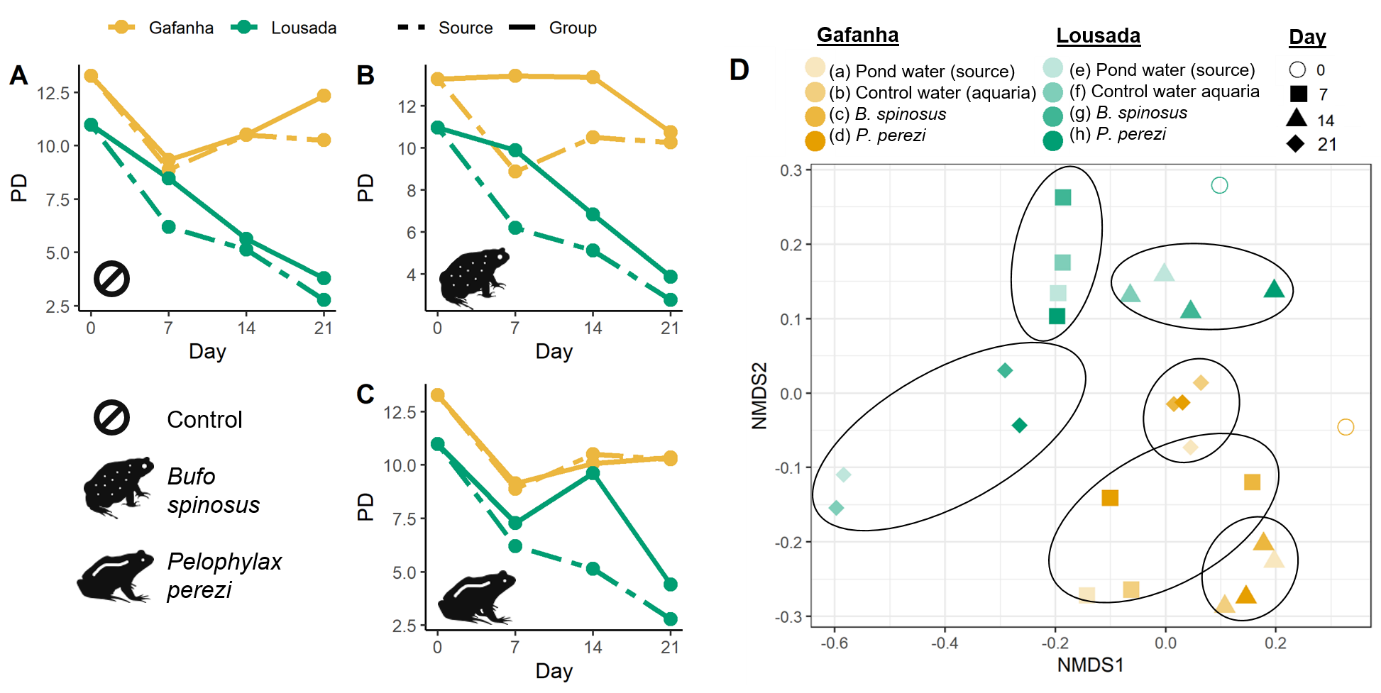
**

**Figure S1** α-diversity (A-C) and β-diversity (D) of the bacterial communities characterizing the water samples used in the experiment over time (day 0 to 21). A-C show the variation in phylogenetic α-diversity observed in the water from each experimental group (solid lines: control (A), *Bufo spinosus* (B) and *Pelophylax perezi* (C)), comparatively to the bacterial communities occurring in the natural waterbodies (dashed line: water source); and β-diversity (D) based on the unweighted unifrac distances among all water samples from each experimental group. Colours represent the two source waters: Gafanha (yellow) and Lousada (green). In D, the different shades distinguish the bacterial communities observed within the experimental groups (control, *B. spinosus*, *P. perezi*) associated to each source water, and symbols represent the different sampling events. The ellipses show the groups with the same water source across days and with tadpoles (darker colours c, d, g, h) and without tadpoles (lighter colours a,b,e,f).

**
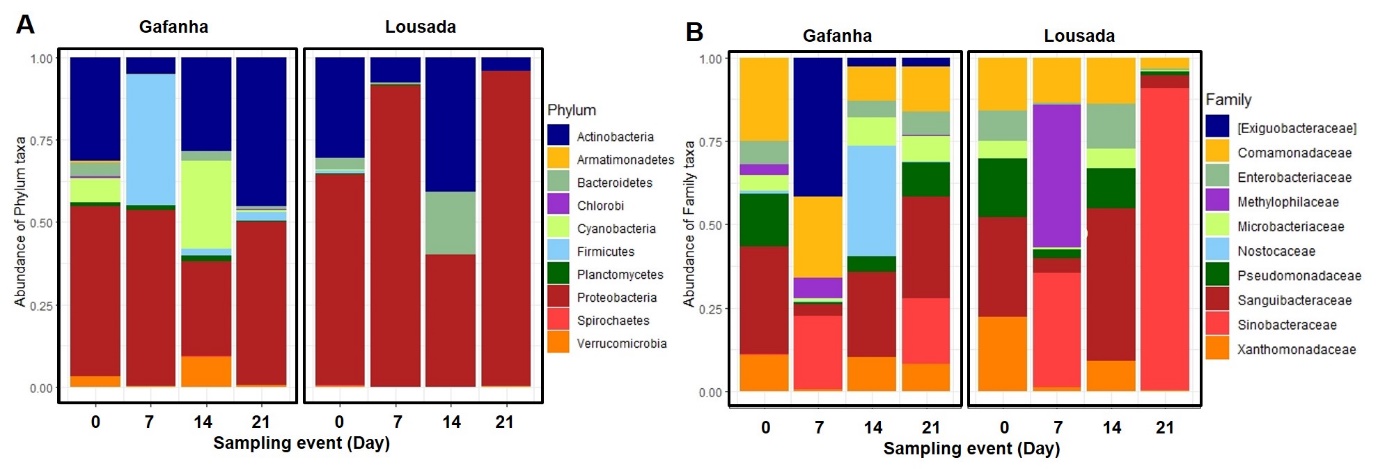
**

**Figure S2** Abundance of the 10 most prevalent bacterial Phyla (A) and Families (B) detected in each water source (Gafanha and Lousada) across the sampling events (Days).


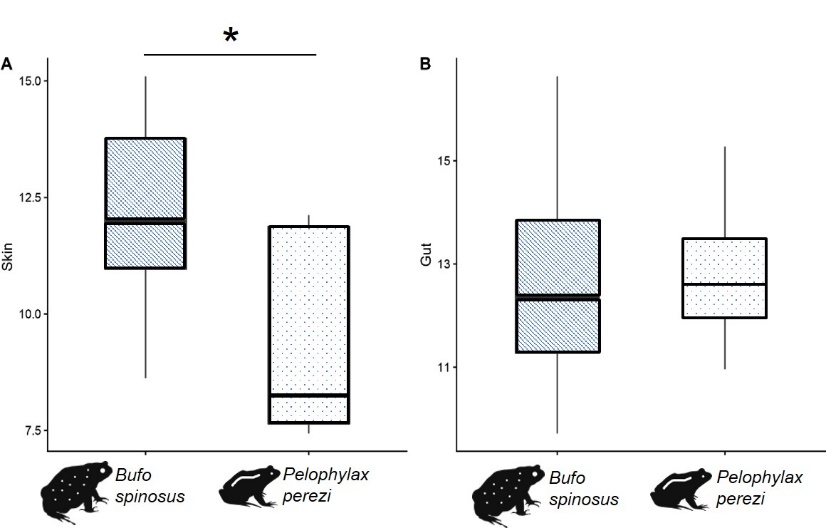


**Figure S3 -** Boxplots of bacterial α-diversity (using the Faith’ phylogenetic diversity metric) detected in the skin (A) and gut (B) of *Bufo spinosus* and *Pelophylax perezi* tadpoles at day 0. Asterisk denotes significant differences between groups.


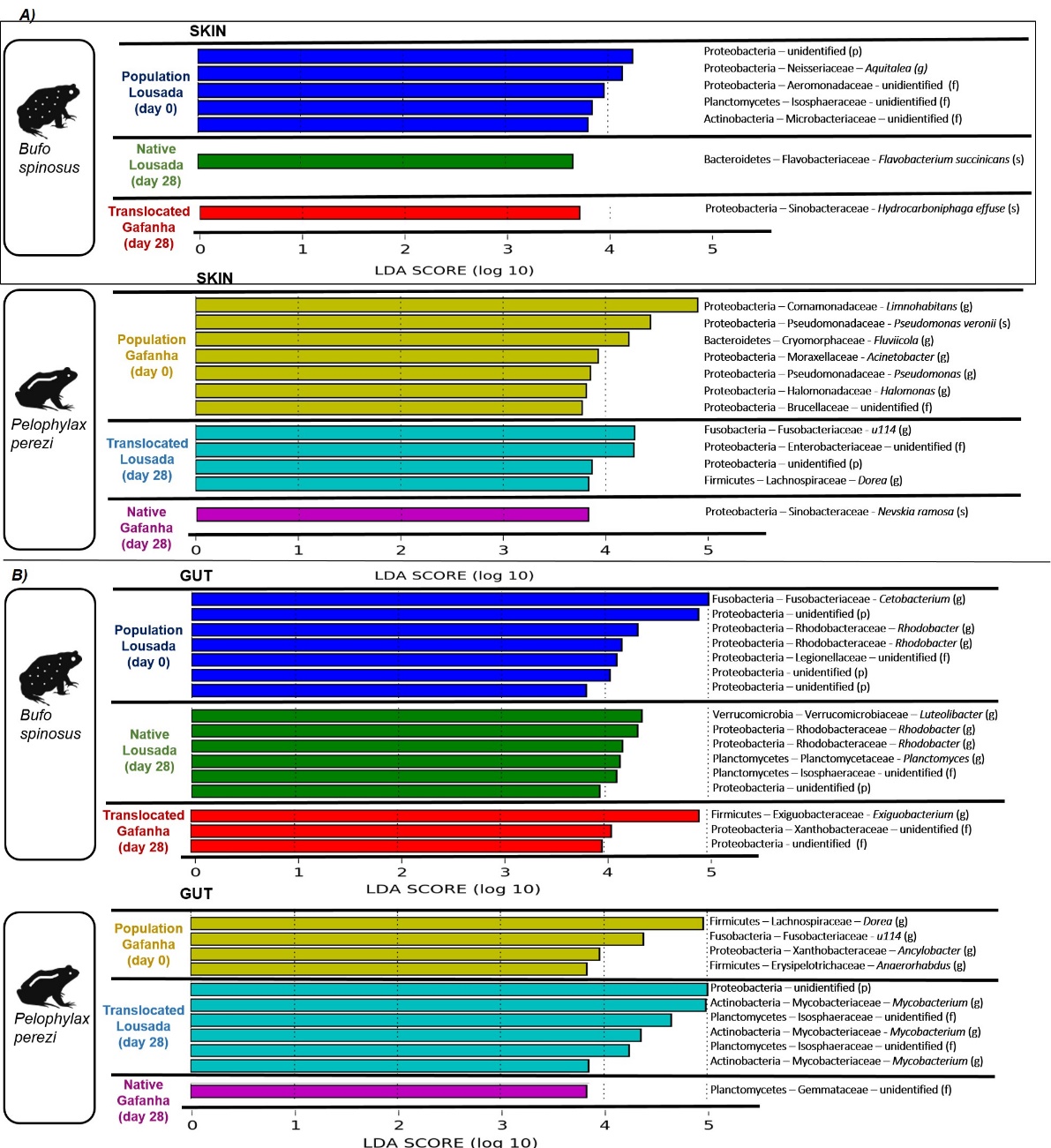


**Figure S4** - Identification of the skin and gut bacteria taxa that were significantly more abundant in each experimental group (host species x water source) at the beginning (Day 0) and at the end (Day 28) of the experiment. A) Skin bacteria; B) Gut bacteria. Bars represent the values for significant LDA scores (LDA>2) of each OTU. Letters between parenthesis following taxa name indicate the taxonomic level of the OTU: p=Phylum, f=Family, g=Genus, s=Species.


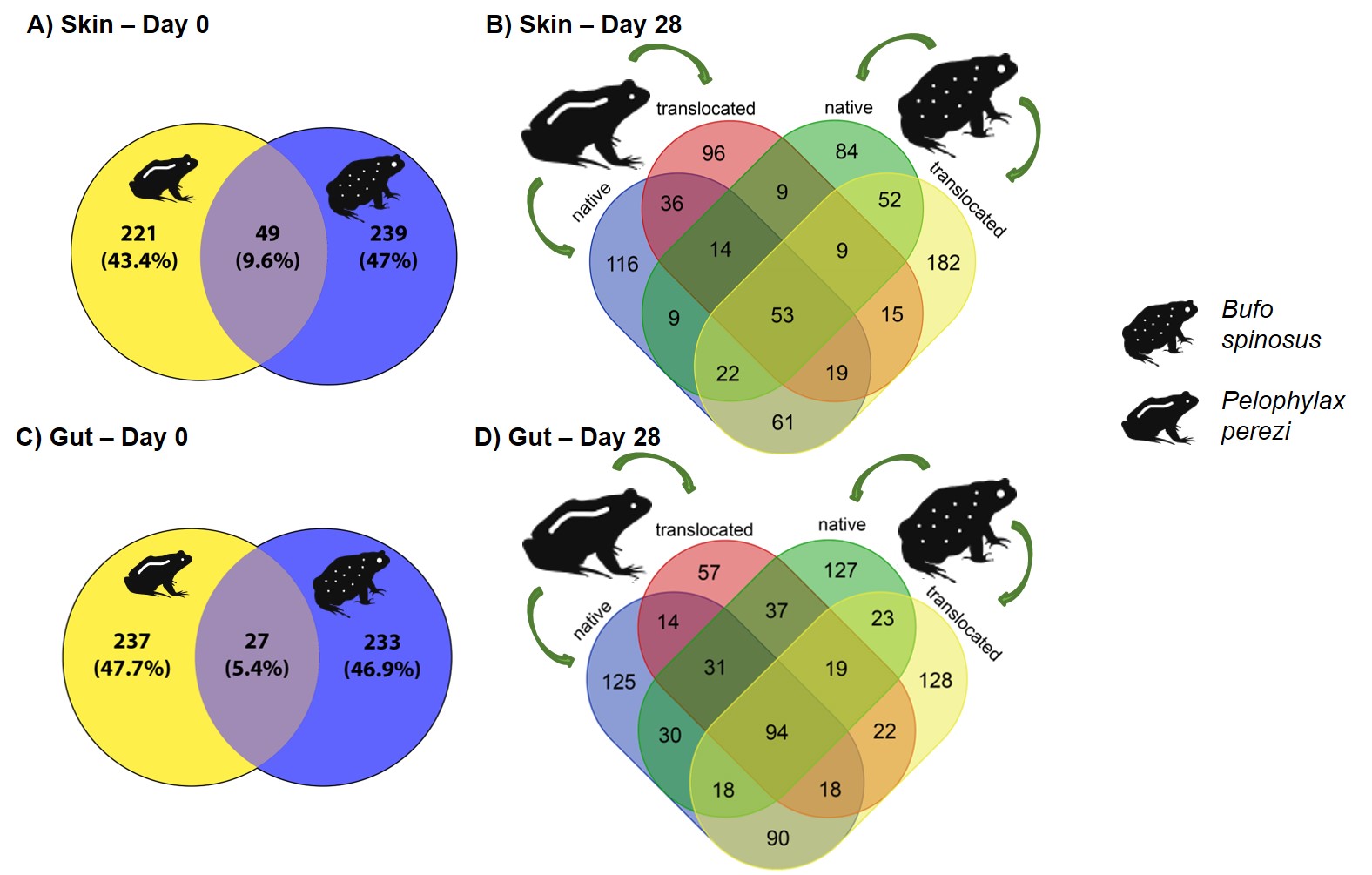


**Figure S5 -** Total of unique and shared bacterial OTUs between tadpoles of *Pelophylax perezi* and *Bufo spinosus* at the beginning of the experiment (day 0) and at the end of the experiment after being exposed to native and translocated waters (day 28): (A) Skin communities at day 0; (B) Skin communities at day 28; (C) Gut communities at day 0; (D) Gut communities at day 28.


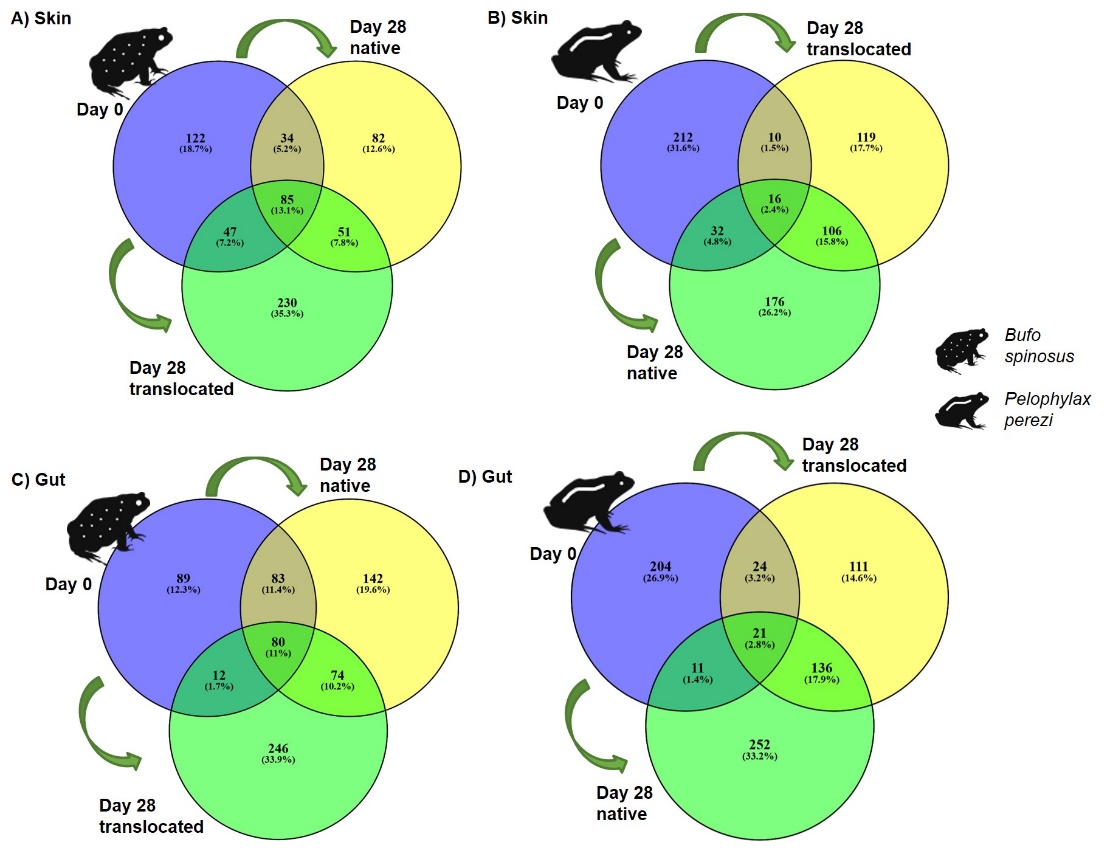


**Figure S6 -** Total of unique and shared bacterial OTUs on the skin and gut of tadpoles of each species at day 0 and day 28 exposed to the two water sources:(A) skin of *Bufo spinosus*; (B) skin of *Pelophylax perezi*; (C) gut of *Bufo spinosus*; (D) gut of *Pelophylax perezi*.

**Table S1** Summary of GLMM statistics used to evaluate the effect of the interaction of source water (native/translocated) and sampling events (Days 0/7/14/21/28) on the phylogenetic α-diversity of (A) skin bacteria of *Bufo spinosus*; (B) skin bacteria of *Pelophylax perezi*; (C) gut bacteria of *B. spinosus*, and (D) gut bacteria of *P. perezi.* Tadpoles of *P. perezi* were collected at Gafanha and tadpoles of *B. spinosus* were collected at Lousada, which correspond to their respective native source water. For each model, we provide the parameter estimates, standard errors (SE), the t value and statistical significance of terms (Pr). Significant values are highlighted in bold.

| SKIN | | | | |
| --- | --- | --- | --- | --- |
| (A) *B. spinosus* | **Estimate** | **Std. Error** | **t value** | **Pr(>\|t\|)** |
| (Intercept) | 12.25757 | 0.56665 | 21.632 | <2e-16*** |
| OriginNative:Day | -0.01787 | 0.03810 | -0.469 | 0.641 |
| OriginTranslocated:Day | 0.16656 | 0.03948 | 4.219 | **9.06e-5**** |
| (B) *P. perezi* | **Estimate** | **Std. Error** | **t value** | **Pr(>\|t\|)** |
| (Intercept) | 8.06193 | 0.71766 | 11.234 | 4.16e-06*** |
| OriginNative:Day | 0.18122 | 0.04877 | 3.716 | **0.00189**** |
| OriginTranslocated:Day | 0.14437 | 0.04704 | 3.069 | **0.00724**** |
| GUT | | | | |
| (C) *B. spinosus* | **Estimate** | **Std. Error** | **t value** | **Pr(>\|t\|)** |
| (Intercept) | 11.35113 | 1.10791 | 10.245 | 0.00105*** |
| OriginNative:Day | 0.07984 | 0.06235 | 1.280 | 0.21808 |
| OriginTranslocated:Day | 0.22716 | 0.06222 | 3.651 | **0.00149**** |
| (D) *P. perezi* | **Estimate** | **Std. Error** | **t value** | **Pr(>\|t\|)** |
| (Intercept) | 12.77742 | 0.57543 | 22.205 | <2e-16*** |
| OriginNative:Day | 0.04058 | 0.03378 | 1.201 | 0.237 |
| OriginTranslocated:Day | -0.05164 | 0.03573 | -1.445 | 0.157 |
| *** p<0.001, ** p<0.01, * p<0.05, ^ns^ p>0.05 | | | | |

**Table S2** Summary of nonparametric permutational multivariate analysis of variance (PERMANOVA; 9,999 permutations) used to evaluate the effects of source water (Gafanha/Lousada), host species (*B. spinosus*/*P. perezi*) and sampling events (Day 0/7/14/21/28) on bacterial composition of (A) Skin; (B) Gut. *P. perezi* tadpoles were collected at Gafanha and *B. spinosus* tadpoles at Lousada, which correspond to their respective native source water. Significant values are highlighted in bold.

| **(A) SKIN** | | | | | | |
| --- | --- | --- | --- | --- | --- | --- |
| **Factors** | **df** | **SumsOfSqs** | **MeanSqs** | **F.Model** | **R2** | **Pr(>F)** |
| **Group** | 1 | 2.786 | 2.78559 | 11.4097 | 0.08093 | **1e-04***** |
| **Group: Origin** | 2 | 1.794 | 0.89712 | 3.6746 | 0.05213 | **1e-04***** |
| **Group: Origin: Day** | 4 | 2.986 | 0.74645 | 3.0574 | 0.08674 | **1e-04***** |
| **Residuals** | 110 | 26.856 | 0.24414 |  | 0.78021 |  |
| **Total** | 117 | 34.421 |  |  | 1.00000 |  |
| **(B) GUT** | | | | | | |
| **Factors** | **df** | **SumsOfSqs** | **MeanSqs** | **F.Model** | **R2** | **Pr(>F)** |
| **Group** | 1 | 1.5686 | 1.56864 | 6.1469 | 0.05975 | **1e-04***** |
| **Group: Origin** | 2 | 2.7119 | 1.35594 | 5.3134 | 0.10330 | **1e-04***** |
| **Group: Origin: Day** | 4 | 2.8326 | 0.70815 | 2.7750 | 0.10790 | **1e-04***** |
| **Residuals** | 75 | 19.1395 | 0.25519 |  | 0.72905 |  |
| **Total** | 82 | 26.2526 |  |  | 1.00000 |  |
| *** p<0.001, ** p<0.01, * p<0.05, ^ns^ p>0.05 | | | | | | |

**Table S3** Identification of core100 OTUs that were associated to each host species (*Bufo spinosus* and *Pelophylax perezi*) and shared among the groups (day0, day28-native, day28-translocated) occurring in the skin or gut.

| Host  species | Phylum | Class | Family | Genus/  Species |
| --- | --- | --- | --- | --- |
| SKIN | | | | |
| *Bufo*  *spinosus* | Actinobacteria | Actinobacteria | Microbacteriaceae | *unidientified* |
|  | Proteobacteria | Gammaproteobacteria | Pseudomonadaceae | *Pseudomonas*  *fragi* |
|  | Actinobacteria | Actinobacteria | Microbacteriaceae | *Microbacterium maritypicum* |
|  | Proteobacteria | Gammaproteobacteria | Enterobacteriaceae | *unidientified* |
|  | Proteobacteria | Gammaproteobacteria | Pseudomonadaceae | *Pseudomonas*  *veronii* |
|  | Proteobacteria | Betaproteobacteria | Comamonadaceae | *unidentified* |
|  | Proteobacteria | Gammaproteobacteria | Xanthomonadaceae | *Stenotrophomonas* |
|  | Proteobacteria | Betaproteobacteria | Comamonadaceae | *Variovorax*  *paradoxus* |
|  | Actinobacteria | Actinobacteria | Sanguibacteraceae | *Sanguibacter* |
| *Pelophylax perezi* | Proteobacteria | Betaproteobacteria | Comamonadaceae | *Variovorax*  *paradoxus* |
|  | Proteobacteria | Betaproteobacteria | Comamonadaceae | *Limnohabitans*  *curvus* |
| *GUT* | | | | |
| *Bufo*  *spinosus* | Proteobacteria | Alphaproteobacteria | unidientified | *unidientified* |
|  | Planctomycetes | Planctomycetia | Gemmataceae | *unidientified* |
|  | Planctomycetes | Planctomycetia | Isosphaeraceae | *unidientified* |
|  | Proteobacteria | Alphaproteobacteria | unidientified | *unidientified* |
|  | Proteobacteria | Alphaproteobacteria | unidientified | *unidientified* |
|  | Actinobacteria | Actinobacteria | Mycobacteriaceae | *Mycobacterium* |
| *Pelophylax perezi* | None | None | None | None |
